# Toxicokinetics, Biotransformation, Excretion, and Tissue Residue of Zearalenone-14-Glucoside in Male Sheep

**DOI:** 10.3390/toxins18090364

**Published:** 2026-08-25

**Authors:** Jiaxin Qin, Qianqian Zhang, Na Ren, Zhenyu Su, Zibin Zheng, Tianzhu Yin, Decheng Suo, Xia Fan, Liwen He, Wei Zhang

**Affiliations:** 1 State Key Laboratory of Animal Nutrition and Feeding, College of Animal Science and Technology, China Agricultural University, Beijing 100193, China; qinjiaxin@cau.edu.cn (J.Q.); zhangqq6@cau.edu.cn (Q.Z.); renna@cau.edu.cn (N.R.); suzhenyu@cau.edu.cn (Z.S.); zhengzibin@cau.edu.cn (Z.Z.); yintianzhu@cau.edu.cn (T.Y.); helw@cau.edu.cn (L.H.); 2 Institute of Quality Standards and Testing Technology for Agricultural Products, Chinese Academy of Agricultural Science, Beijing 100081, China; suodecheng@caas.cn (D.S.); fanxia@caas.cn (X.F.)

**Keywords:** zearalenone-14-glucoside, masked mycotoxin, ruminants, toxicokinetic, tissue residue, rumen hydrolysis

## Abstract

Zearalenone-14-glucoside (Z14G) is a common modified mycotoxin attracting considerable attention due to its high occurrence in feed and food, and its potential to transform into the parent form zearalenone (ZEN) in vivo. However, the toxicokinetic data in ruminants remain scarce. In this study, male sheep (*n* = 5) were orally administered a single dose of Z14G at 2 mg·kg^−1^ BW. Serum, rumen fluid, urine, and fecal samples were collected at designated time points, and tissue samples were obtained at slaughter 168 h post-dosing. Concentrations of Z14G, ZEN, α-zearalenol (α-ZOL), β-zearalenol (β-ZOL), α-zearalanol (α-ZAL), β-zearalanol (β-ZAL), zearalanone (ZAN), and zearalenone-14-sulfate (Z14S) in these samples were determined using liquid chromatography coupled with tandem quadrupole linear ion trap mass spectrometry (LC-Qtrap-MS/MS), so as to explore the absorption, distribution, biotransformation, excretion patterns, and tissue residue profiles of Z14G. The results showed that Z14G was rapidly hydrolyzed in the rumen, with multiple metabolites detected within 1 h. Among these, β-ZOL exhibited concentrations of 154.75 ± 55.66 ng·mL^−1^, followed by ZEN 83.56 ± 33.36 ng·mL^−1^. In serum, ZEN, β-ZOL, and Z14G all reached peak concentrations at 30 min, with values of 20.25 ± 2.55, 3.01 ± 0.59 and 2.15 ± 0.78 ng·mL^−1^, respectively. The half-lives of all three analytes were less than 3 h, with Z14G eliminated the fastest (t_1/2_ = 0.94 ± 0.31 h) and ZEN the slowest (t_1/2_ = 2.91 ± 1.49 h). The total excretion of Z14G and its metabolites in feces and urine accounted for 0.90 ± 0.14% and 0.04 ± 0.01%, respectively. Z14G was not detected in any tissues, but ZEN was detected (1.38–3.61 ng·g^−1^). As the first systematic toxicokinetic investigation of Z14G in ruminants, this study provides critical data for food safety risk assessment of Z14G, and offers a theoretical basis for controlling Z14G contamination in ruminant feed.

## 1. Introduction

Zearalenone-14-glucoside (Z14G) is a masked mycotoxin formed during plant metabolism of zearalenone (ZEN). Its structural modification allows it to escape conventional detection, potentially leading to an underestimation of its true contamination levels in food and feed. Numerous worldwide contamination surveys have confirmed that Z14G is widely present in cereal-based foods, grain by-products, and feed, with detection rates ranging from 6% to 60% and concentrations reaching up to 369 μg·kg^−1^, and that masked and free mycotoxins frequently co-occur [1,2,3,4,5,6]. Occasionally, animals have been reported to show mycotoxicosis after consuming feed contaminated with mycotoxin levels below the established toxic dose. This phenomenon may be associated with the additive toxicity of masked and free toxins [7]. Current feed regulatory frameworks do not account for modified forms of zearalenone such as Z14G, creating uncertainties for livestock-health risk assessment.

In vitro experiments demonstrate that Z14G undergoes complete glycosidic bond cleavage upon incubation with human colonic microbiota for 30 min, resulting in the liberation of free ZEN [8]. Z14G remains stable in simulated upper digestive fluids (including artificial saliva, gastric juice, duodenal juice, and bile) but can be metabolized by the gut microbiota, releasing free parent toxins [9]. In liver microsomes from rats, chickens, pigs, goats, cows, and humans, Z14G can be hydrolyzed to ZEN, α-zearalenol (α-ZOL), β-zearalenol (β-ZOL), and other metabolites [10]. Notably, α-ZOL exhibits markedly higher estrogenic activity than ZEN and demonstrates greater toxicity than ZEN in both genotoxicity and immunotoxicity assays [7]. The European Food Safety Authority (EFSA) has stated that α-ZOL and α-zearalanol (α-ZAL) possess higher estrogenic potency than ZEN, and that risk assessment should account for the cumulative toxicity of masked ZEN forms following their in vivo transformation to the parent toxin [11].

Although in vitro studies provide important insights into the potential toxicity of Z14G, in vivo studies can integrate complex physiological factors such as enzymatic systems, microbial interactions, and enterohepatic circulation. Current in vivo research on Z14G is mainly focused on monogastric animals. Studies in rats show that Z14G has extremely low oral bioavailability (2.11–8.89%) [12,13]. Following oral administration, it is rapidly converted in vivo to ZEN and further metabolized to α-ZOL, β-ZOL and other metabolites, which are detectable in serum, tissues, urine, and feces [12,13]. Furthermore, after degradation to ZEN in the rat intestine, Z14G induces toxicity by altering gut microbial abundance and disrupting the intestinal lipid–immune axis, with greater toxicity to the liver and ovaries compared to ZEN alone [14]. Studies in piglets show that Z14G is efficiently hydrolyzed to ZEN and further converted to α-ZOL in the proximal gastrointestinal tract, with an absorbed fraction of 61–94% [15,16,17]. ZEN and its metabolites may prolong their residence time in vivo through enterohepatic circulation [15,16,17]. ZEN is widely distributed in tissues and can cross the blood–brain barrier to enter the brain [7]. Although its concentrations in hormone-secreting organs such as the uterus and ovaries are low, even low levels can lead to severe dysfunction [7]. Collectively, these findings indicate that the toxicity of Z14G primarily stems from its conversion to ZEN and subsequent metabolites, with marked interspecies differences.

In contrast to monogastric animals, ruminants possess a unique rumen ecosystem. Rumen microorganisms can efficiently convert ZEN to the more toxic α-ZOL and the less toxic β-ZOL, thereby modulating the overall toxicity of ZEN [18]. Moreover, the ability of rumen microbes to hydrolyze glycosidic bonds suggests that Z14G may be rapidly hydrolyzed in the rumen. However, no studies have yet reported on the toxicokinetics, tissue distribution, or excretion patterns of Z14G in ruminants. We therefore hypothesized that following oral administration, Z14G would be hydrolyzed in the rumen, with its metabolites appearing in the systemic circulation and being eliminated via fecal and urinary excretion. Given their role as the primary fattening animals in the sheep industry, male sheep hold significant research value. In this study, male sheep were used as a model and received a single oral dose of Z14G (2 mg·kg^−1^ BW). Serum, rumen fluid, urine, and fecal samples were collected at various time points, and tissue samples were collected at slaughter 168 h post-dosing. Concentrations of Z14G and its metabolites, including ZEN, α-ZOL, β-ZOL, α-ZAL, β-zearalanol (β-ZAL), zearalanone (ZAN), and zearalenone-14-sulfate (Z14S), were determined using liquid chromatography coupled with tandem quadrupole linear ion trap mass spectrometry (LC-Qtrap-MS/MS). This study provides the first systematic characterization of Z14G ruminal hydrolysis, toxicokinetics, and tissue residue profiles in ruminants, thereby offering a scientific basis for the risk assessment of Z14G in feed.

## 2. Results

### 2.1. LC-MS/MS Method Validation

**Figure 1:**
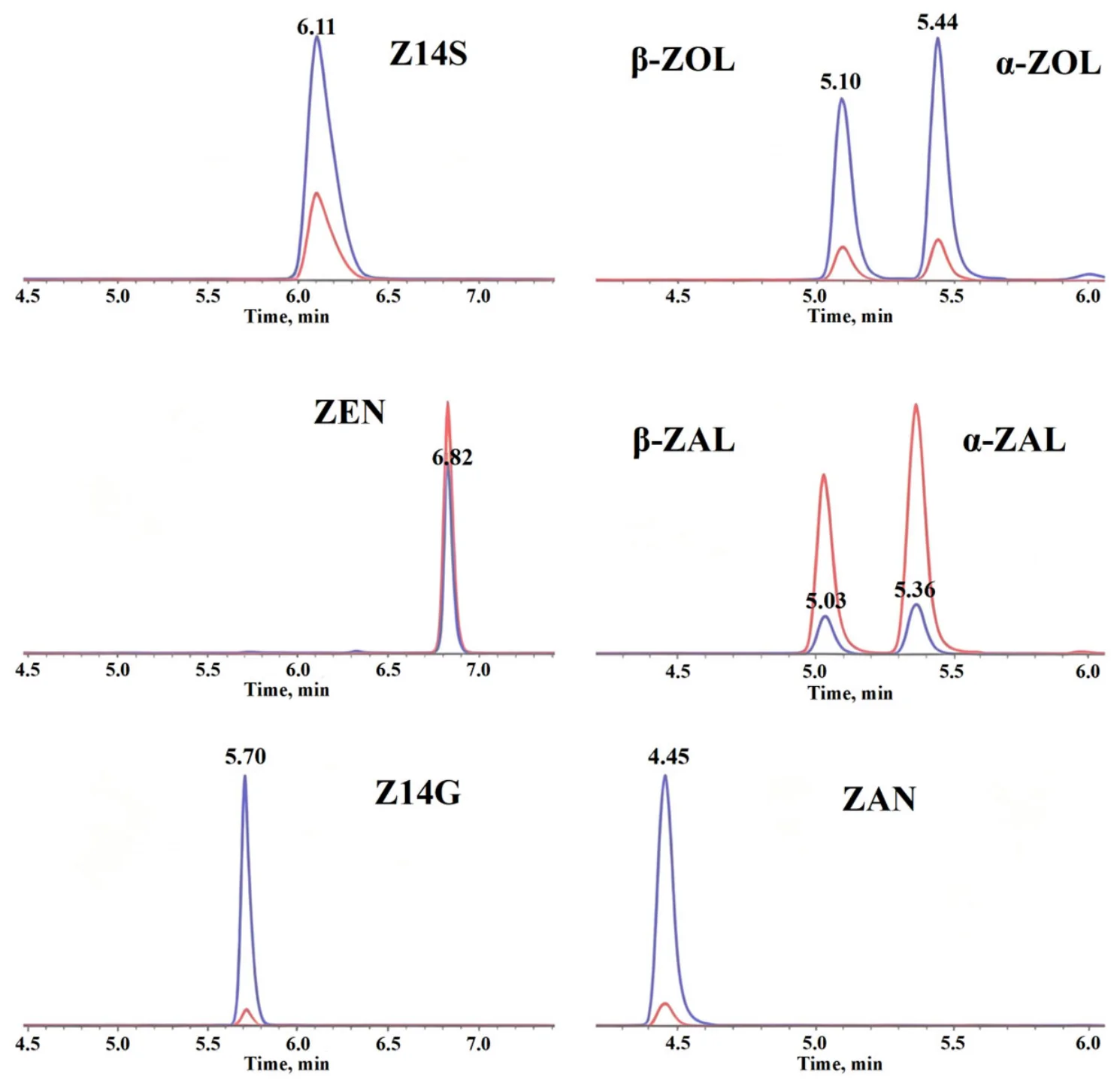
Chromatogram of Z14G and its metabolites. Note: Red traces represent qualitative ion transitions; blue traces represent quantitative ion transitions.

The LC-Q-trap-MS/MS method was used in this study to quantify Z14G and its seven metabolites (ZEN, ZAN, α-ZOL, β-ZOL, α-ZAL, β-ZAL, and Z14S) [19]. Typical total ion chromatograms (Figure 1) showed good separation of all analytes in different matrices, with clear and stable retention times and no significant matrix interference or co-eluting peaks, indicating good specificity. Correlation coefficient (R^2^ ≥ 0.9990) was observed for all analytes across the concentration range of 0.5–100 μg·L^−1^ (Table 1). The limits of detection (LOD) and quantification (LOQ) were in the ranges of 0.09–0.21 μg·L^−1^ and 0.3–0.5 μg·L^−1^, respectively, demonstrating high sensitivity for trace residue analysis. Validation parameters (matrix effect, intra-assay and inter-assay recovery, and precision) were determined for different matrices with full datasets summarized in Table S1. Matrix effects ranged from 6.0% to 22.4%, with intra-assay recoveries of 90.5–99.9% (RSD ≤ 13.3%) and inter-assay recoveries of 89.7–99.7% (RSD ≤ 15.0%). These results met the acceptance criteria for residue analysis, demonstrating that the method is accurate, reliable, and suitable for subsequent in vivo sample analysis.

### 2.2. Toxicokinetic Parameters of Z14G in the Serum of Sheep

Following oral exposure, Z14G was absorbed into the bloodstream of sheep. Z14G, ZEN, and β-ZOL were detected in serum samples. As shown in Table 2, the serum concentrations of β-ZOL, ZEN, and Z14G all reached their peak (C_max_) at 0.50 ± 0.00 h after oral administration. The elimination half-lives were 2.79 ± 0.92, 2.91 ± 1.49, 0.94 ± 0.31 h, and the apparent volumes of distribution were 972.91 ± 124.35, 397.00 ± 175.26, 1037.13 ± 194.93 L·kg·BW^−1^, respectively.

### 2.3. Serum Concentration of Z14G

**Figure 2:**
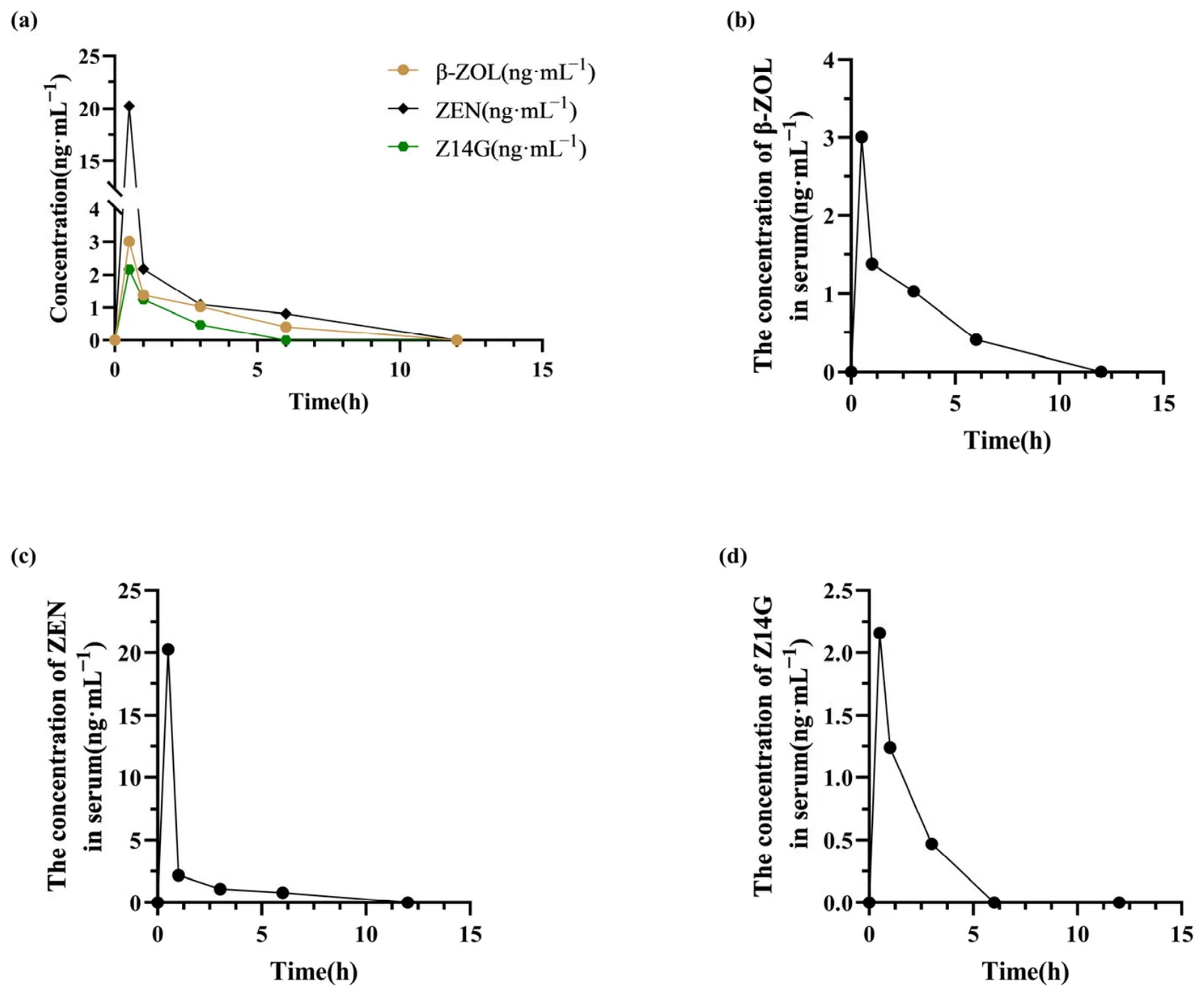
Serum concentration–time profiles for Z14G in male sheep after oral gavage of 2 mg·kg^−1^·BW, *n* = 5. (**a**) Serum concentration–time curves of ZEN, β-ZOL and Z14G in male sheep. (**b**) Serum concentration–time profile of β-ZOL. (**c**) Serum concentration–time profile of ZEN. (**d**) Serum concentration–time profile of Z14G. Note: All metabolite concentrations at time points after 12 h were below the LOD.

As shown in Figure 2, after a single oral administration, the serum concentrations of Z14G, ZEN and β-ZOL all increased rapidly, reaching peak levels at 0.50 ± 0.00 h before declining gradually. ZEN was first detected at 30 min post-dosing. Z14G could not be detected at 6 h after oral gavage, while ZEN and β-ZOL fell below the detection limit at 12 h.

### 2.4. Local Kinetic Characteristics of Z14G in the Rumen of Sheep

**Figure 3:**
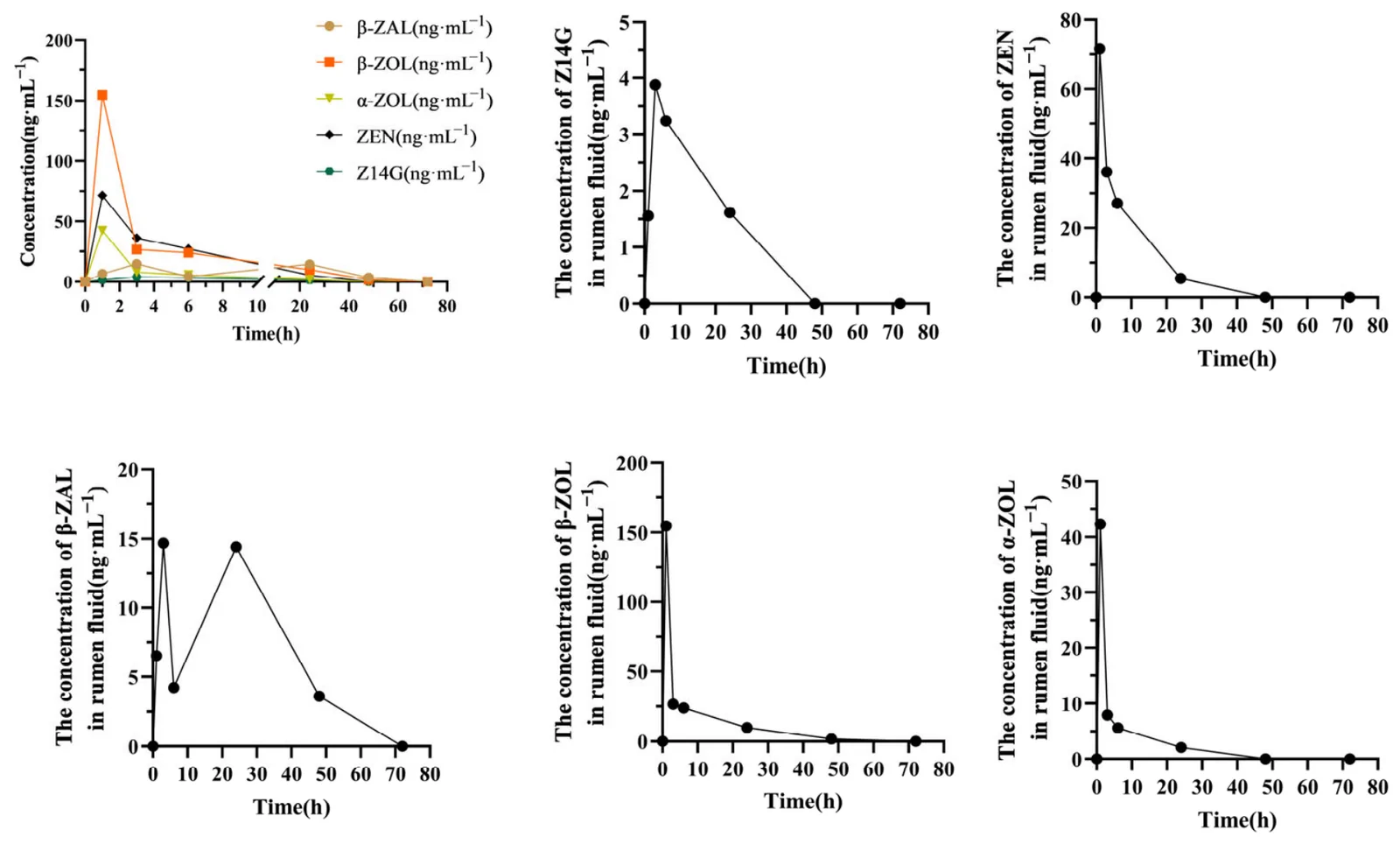
Changes in concentrations of Z14G and its metabolites in sheep rumen fluid over time (*n* = 5). Note: All metabolite concentrations at time points after 48 h were below the LOD.

Following oral exposure to 2 mg·kg^−1^·BW Z14G, Z14G and its metabolites ZEN, β-ZOL, α-ZOL and β-ZAL were detectable in the rumen fluid of male sheep. As shown in Figure 3 and Table 3, the time to maximum concentration (T_max_) of β-ZOL, α-ZOL, and ZEN was 1.00 h, which was earlier than that of β-ZAL and Z14G (both 3.00 h). For the measured ruminal analytes, the C_max_ of β-ZOL (154.75 ± 55.66 ng·mL^−1^) was the highest, followed by ZEN (83.56 ± 33.36 ng·mL^−1^), α-ZOL (42.30 ± 27.66 ng·mL^−1^), and β-ZAL (21.71 ± 5.55 ng·mL^−1^), while the C_max_ of Z14G was the lowest of only 4.16 ± 0.69 ng·mL^−1^. The trends of the area under the curve in the rumen (AUC_rum_) and C_max_ were consistent within 0–72 h. β-ZOL had the highest cumulative exposure (778.85 ± 254.68 ng·mL^−1^·h), followed by ZEN (568.02 ± 257.79 ng·mL^−1^·h), β-ZAL (437.24 ± 219.39 ng·mL^−1^·h), and α-ZOL (161.54 ± 76.20 ng·mL^−1^·h), with Z14G having the lowest (38.81 ± 27.08 ng·mL^−1^·h). The elimination kinetic parameters are listed in Table 3. ZEN had the shortest elimination half-life at 7.44 ± 2.31 h. The elimination half-lives of β-ZOL (11.90 ± 3.33 h) and α-ZOL (10.60 ± 3.16 h) were relatively long and comparable to each other. Among these compounds, the calculated apparent absorption efficiency (F_rum_) (Table 4) showed that Z14G had the highest F_rum_ (10.74 ± 5.68%), higher than those of ZEN (3.50 ± 1.47%) and β-ZOL (1.08 ± 0.71%).

As shown in Figure 3, the concentrations of ZEN, β-ZOL and α-ZOL increased rapidly at 1 h post-administration. Among them, β-ZOL reached its peak concentration first at 154.75 ng·mL^−1^. By contrast, the concentration of Z14G was extremely low at this time point, and its peak concentration of 3.88 ng·mL^−1^ was observed at 3 h, followed by a gradual decline thereafter. The concentration of ZEN decreased continuously after peaking at 1 h and dropped below the limit of detection at 48 h. The concentration–time curve of β-ZAL exhibited a bimodal pattern, with two peaks appearing at 1 h and 24 h. By 72 h, Z14G, ZEN, and all metabolites were no longer detectable in the rumen fluid, and they remained undetectable at 168 h.

### 2.5. Recovery of Z14G and Its Metabolites Excreted in Feces and Urine

**Figure 4:**
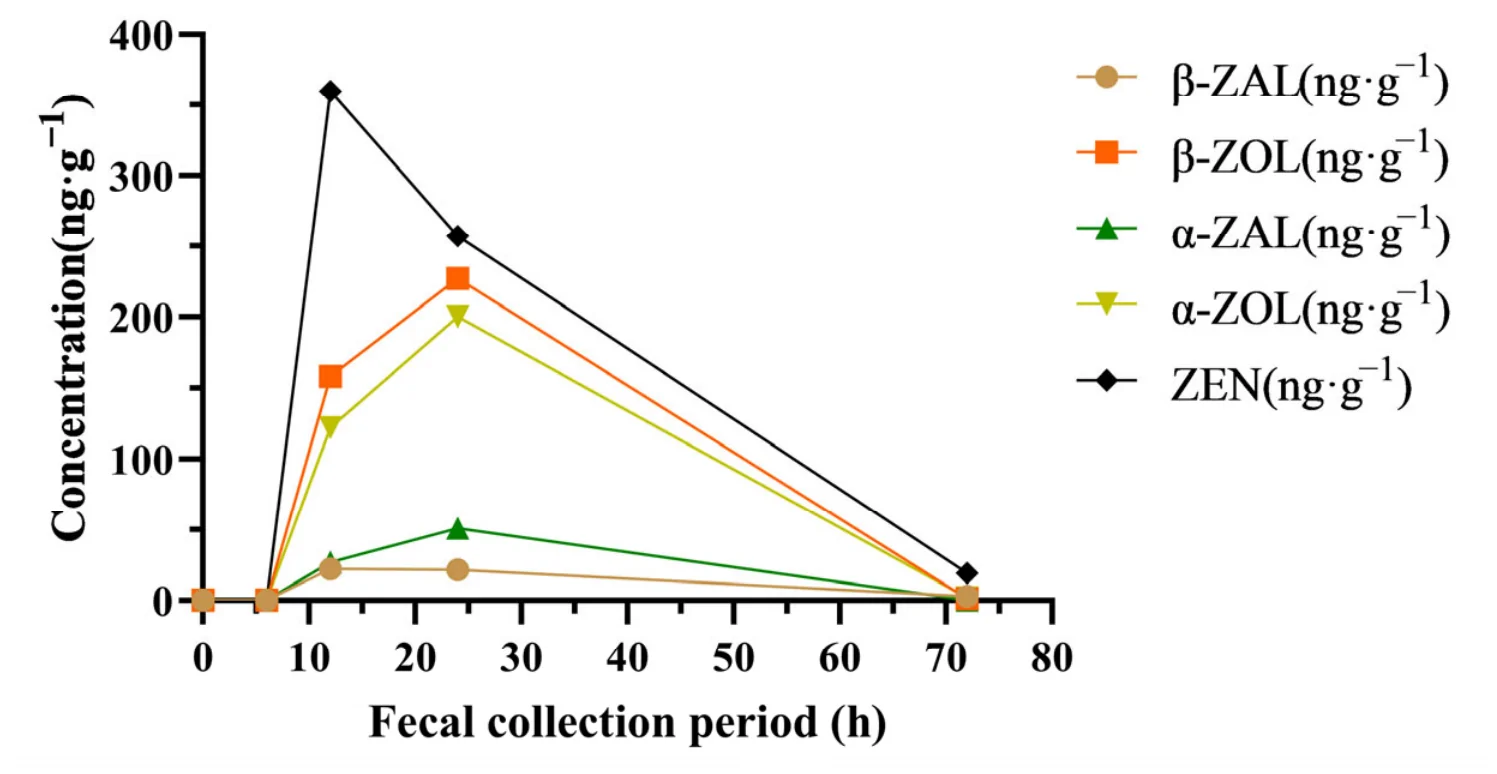
Excretion profiles of Z14G metabolites in sheep feces following a single oral administration (*n* = 5). Note: Data represent metabolite concentrations (ng·g^−1^) in cumulative fecal samples collected at each interval. Z14G, Z14S, and ZAN were not detected in any fecal samples. All metabolite concentrations at time points after 72 h were below the LOD.

**Figure 5:**
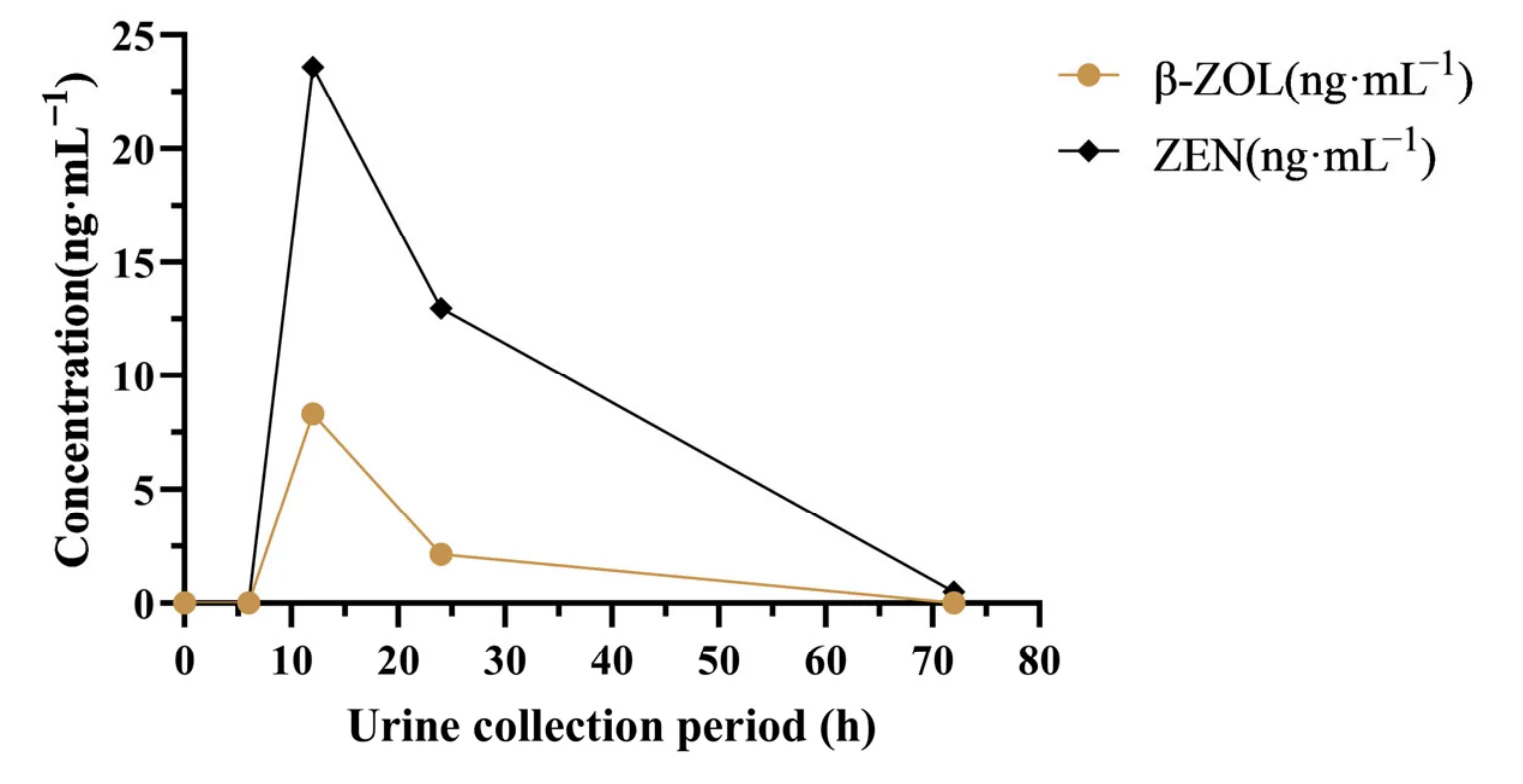
Excretion profiles of Z14G metabolites in sheep urine following a single oral administration (*n* = 5). Note: Data represent metabolite concentrations (ng·mL^−1^) in cumulative urine samples collected at each interval. β-ZAL, α-ZAL, α-ZOL, Z14G, Z14S, and ZAN were not detected in any urine samples. All metabolite concentrations at time points after 12 h were below the LOD.

Throughout the entire sampling period, Z14G was not detected in any fecal or urine samples (Figure 4 and Figure 5). In fecal samples, β-ZAL, β-ZOL, α-ZAL, α-ZOL, and ZEN were first detected between 6 and 12 h post-oral administration. Of the measured metabolites, ZEN exhibited the highest observed concentration, reaching a peak concentration of 359.92 ng·g^−1^, followed by β-ZOL (158.29 ng·g^−1^) and α-ZOL (122.00 ng·g^−1^). The concentrations of α-ZAL and β-ZAL were relatively low. Between 12 and 24 h post-administration, the concentrations of β-ZOL and α-ZOL reached their peak concentrations of 227.33 ng·g^−1^ and 200.29 ng·g^−1^, respectively. In contrast, the ZEN concentration decreased during this period, falling to 257.3 ng·g^−1^. From 24 to 72 h, the levels of all metabolites decreased considerably. Only trace amounts of β-ZAL, β-ZOL, α-ZOL, and ZEN remained detectable, whereas α-ZAL was no longer quantifiable. By 168 h, all target metabolites had completely disappeared from the fecal samples.

In urine samples collected between 6 and 12 h post-administration, ZEN and β-ZOL were detected at concentrations of 23.6 ng·mL^−1^ and 8.3 ng·mL^−1^, respectively. Between 12 and 24 h, the concentrations of both metabolites exhibited a sharp decline. Between 24 and 72 h, only trace amounts of ZEN (0.49 ng·mL^−1^) were detectable, while β-ZOL had completely disappeared. By 168 h, all target metabolites were entirely absent from the urine samples.

Feces and urine are the two major routes of toxin elimination in animals and humans. As shown in Table 5, the total intake of Z14G was 72.12 ± 2.41 mg after a single oral administration in male sheep (2 mg·kg^−1^ BW). The total metabolites (ZEN, β-ZOL, α-ZOL, α-ZAL, and β-ZAL) excreted via feces accounted for 0.90 ± 0.14% of the intake, while the total metabolites (ZEN and β-ZOL) excreted via urine accounted for 0.04 ± 0.01% of the intake. Based on these data, the apparent non-fecal recovery of Z14G in sheep was 99.10 ± 0.14%, calculated as 100% minus the percentage of intake recovered as free metabolites in feces. These findings indicate that feces represent the major excretion route for the measured free metabolites of Z14G in male sheep.

### 2.6. Tissue Distribution

The tissue distribution results at 168 h post-administration (Table 6) showed that only ZEN residues were detected in the examined organs, while Z14G and its other metabolites (β-ZAL, β-ZOL, α-ZAL, α-ZOL, Z14S, ZAN) were not detected. ZEN was detected in all examined tissues from the same two out of five sheep, with one of these two animals also testing positive in bile. ZEN was widely distributed across various tissues, with relatively higher residual levels observed in the heart (3.61 ng·g^−1^) and lung (3.44 ng·g^−1^), and the lowest level in the liver (1.38 ng·g^−1^). Detectable residues were also found in the epididymis (3.05 ng·g^−1^), spleen (3.04 ng·g^−1^), testis (2.32 ng·g^−1^), kidney (2.28 ng·g^−1^), and bile (1.65 ng·mL^−1^).

## 3. Discussion

Under the reversed-phase chromatographic conditions used in this study, the eight target compounds were effectively separated based on their structural characteristics. Their retention times were as follows: ZAN (4.45 min), β-ZAL (5.03 min), β-ZOL (5.10 min), α-ZAL (5.36 min), α-ZOL (5.44 min), Z14G (5.79 min), Z14S (6.11 min) and ZEN (6.62 min). ZAN features saturation at the 1′, 2′ double bond, with its 7′ ketone group reduced to a hydroxyl moiety. On this structural basis, ZAL and ZOL undergo further reduction at the C11′ ketone [7]. All these reductive metabolites exhibit higher polarity than the parent ZEN, thus displaying weaker retention on reversed-phase columns and eluting earlier. Among stereoisomers with identical core skeletons, β-forms elute prior to their corresponding α-forms, which aligns with the polarity differences arising from their steric configurations [7]. Although Z14G and Z14S bear hydrophilic glycoside or sulfate moieties, their macrocyclic lactone skeleton still conferred relatively strong reversed-phase retention, with elution times later than most reductive metabolites but earlier than ZEN. ZEN, lacking hydrophilic substituents, exhibited the strongest hydrophobicity and thus the latest elution. The stable retention times and the absence of co-eluting interference further demonstrated the good selectivity and specificity of this method.

Following oral exposure, Z14G was rapidly hydrolyzed in the digestive tract of sheep and quickly absorbed into the blood. The C_max_ of ZEN (20.25 ± 2.55 ng·mL^−1^) was much higher than that of Z14G (2.15 ± 0.78 ng·mL^−1^), suggesting extensive pre-absorption first-pass hydrolysis of Z14G. Correspondingly, the elimination half-life (T_1/2_) of Z14G (0.94 ± 0.31 h) was significantly shorter than that of ZEN (2.91 ± 1.49 h) and β-ZOL (2.79 ± 0.92 h). The clearance of Z14G (412.12 ± 113.12 L·kg^−1^·h^−1^) was much higher than that of ZEN (96.58 ± 7.63 L·kg^−1^·h^−1^) and β-ZOL (222.64 ± 24.68 L·kg^−1^·h^−1^), confirming rapid clearance of the Z14G parent compound in vivo. This result is consistent with the findings of Lu et al. [12] in rats, where ZEN peaked at 0.25–0.50 h after intragastric administration of Z14G, with ZEN concentrations higher than those of Z14G. Catteuw et al. [15] also confirmed that Z14G was almost completely hydrolyzed to ZEN before reaching the portal circulation in pigs. These results suggest that Z14G exhibits prodrug-like characteristics across different mammalian species.

In this study, β-ZOL was the only reductive metabolite detected in serum, while α-ZOL, α-ZAL and β-ZAL were not identified (Figure 2). The C_max_ of β-ZOL was 3.01 ± 0.59 ng·mL^−1^, approximately one-seventh of that of ZEN. This metabolic profile is closely related to species specificity. Malekinejad et al. [20] reported that 3β-hydroxysteroid dehydrogenase (3β-HSD) activity predominates in ruminant livers, primarily reducing ZEN to β-ZOL with low estrogenic activity, while 3α-HSD dominates in the liver of pigs and humans and yields large amounts of highly estrogenic α-ZOL. Dong et al. [21] detected β-ZOL as the main metabolite in the plasma of goats after intravenous injection of ZEN. In contrast, Lu et al. [12] in rats and Catteuw et al. [15] in pigs both detected α-ZOL as the major reductive metabolite. Qu et al. [22] found only ZEN and no ZOL metabolites in the plasma of female Dezhou donkeys after oral administration of ZEN, suggesting that ZEN reductase activity may be relatively low in equine animals. In summary, the metabolic profile characterized by predominant β-ZOL production in sheep is consistent with the enzymatic basis of dominant hepatic 3β-HSD activity in ruminants.

Regarding elimination kinetics, the AUC_last_ of Z14G (3.10 ± 0.61 ng·mL^−1^·h) was only about one-fifth that of ZEN (16.78 ± 1.08 ng·mL^−1^·h), indicating that the systemic exposure of Z14G was extremely limited. This pattern was consistent with previous data from Lu et al. [12] obtained in rat models, who reported that the fast clearance of Z14G was attributed to its elevated polarity and reduced binding ability to serum albumin. Catteuw et al. [15] found that Z14G similarly underwent rapid systemic hydrolysis in pigs, with a short residence time in plasma and a significantly faster elimination rate than ZEN. Using fluorescence spectroscopy combined with molecular docking, Faisal et al. [23] confirmed that ZEN and its ZOL metabolites exhibited markedly stronger binding affinity for human serum albumin than Z14G, which elucidated the molecular basis for the observed variations in elimination half-life. Notably, β-ZOL presented the maximum AUC_INF_ (8.21 ± 2.16 ng·mL^−1^·h) and the longest elimination half-life (2.79 ± 0.92 h) among Z14G and its metabolites. It was identified as the major metabolite of Z14G in sheep at the terminal elimination phase with prolonged in vivo exposure. Despite its much lower estrogenic activity (RPF = 0.2) compared with α-ZOL (RPF = 60) [11], long-term retention of β-ZOL in the body could continuously stimulate estrogen-sensitive tissues at a low level and lead to potential chronic toxicity.

The apparent volumes of distribution (Vz/F) of Z14G, ZEN, and β-ZOL were 1037.13 ± 194.93, 397.00 ± 175.26, and 972.91 ± 124.35 L·kg^−1^, respectively. All values were considerably higher than the total body water of sheep (approximately 0.6 L·kg^−1^), indicating that these compounds were not restricted to the blood circulation but extensively penetrated into peripheral tissues. Qu et al. [22] determined that the apparent volume of distribution of ZEN in Dezhou donkeys was 216.17 L·kg^−1^, which also observed a large distribution volume. They suggested that ZEN exhibited extensive tissue distribution and a long tissue residence time in vivo. Lu et al. [12] conducted tissue distribution experiments in rats administered Z14G by gavage and further confirmed the above results. ZEN was detectable in all 12 types of rat tissues, with the highest concentration in the gastrointestinal tract and remarkable levels found in the liver, kidneys and reproductive organs. These results indicated that Z14G and its metabolites exhibit extensive tissue distribution across ruminants, equines, and rodents, suggesting that these compounds may accumulate in target organs, posing a potential persistent toxicological risk.

Beyond systemic blood kinetic profiles, the rumen serves as the major metabolic site for xenobiotics in ruminants, where Z14G undergoes transformation pathways distinct from those in monogastric animals.

The rumen is a complex and dynamic microecosystem. The concentrations of Z14G and its metabolites are affected by microbial metabolism, digesta emptying, enterohepatic circulation and other factors. Some metabolites presented bimodal concentration curves, which did not meet the fitting assumptions of compartmental models. Therefore, non-compartmental analysis (NCA) was adopted to determine the local toxicokinetic parameters in the rumen. Following oral exposure to 2 mg·kg^−1^·BW Z14G, Z14G underwent rapid deglycosylation after entering the rumen to produce ZEN, which was subsequently further reduced to ZOL metabolites by rumen microorganisms. Gratz et al. [9] found that Z14G began to be hydrolyzed within 30 min and over 97% was transformed within 4 h by human gut microbiota. Kiessling et al. [24] reported that rumen microorganisms could convert more than 90% of ZEN into α-ZOL and β-ZOL, which serves as the first line of defense against ZEN toxicity in ruminants. Guo [25] further confirmed via an in vitro rumen fermentation experiment in dairy cows that ZEN could be degraded by 34–58% within 48 h, and the degradation mainly occurred in the first 6 h. In summary, Z14G underwent a two-step metabolic transformation in the rumen, consisting of initial hydrolysis followed by reduction, with rumen microorganisms playing a critical barrier role in this process.

The results indicate strong reductive conversion of ZEN to β-ZOL in the rumen, establishing β-ZOL as the main exposure component in the rumen. In this study, Z14G was used as the test substrate. The C_max_ and AUC of β-ZOL in ovine ruminal fluid were significantly higher than those of α-ZOL. This finding differed from the results of in vitro rumen experiments conducted by Kiessling et al. [24] and Seeling et al. [26]. They reported that rumen microorganisms preferentially converted ZEN into α-ZOL, with β-ZOL as a minor metabolite. The predominance of β-ZOL over α-ZOL in ruminal fluid reflects the unique rumen microbial metabolic characteristics of sheep under in vivo conditions, which differ substantially from those observed in in vitro rumen simulation systems, likely due to the absence of continuous substrate supply, digesta flow, and systemic absorption in the latter. Debevere et al. [27] also demonstrated that ruminal pH markedly affected the transformation efficiency of ZEN, and an acidic rumen environment could inhibit this metabolic process. Rumen pH further modulates metabolite formation. Near neutral pH under regular feeding favors β-reduction pathways, whereas acidic conditions suppress ZEN biotransformation and reshape isomer distribution [27]. All sheep were maintained on a standardized twice-daily feeding schedule throughout the sampling period, with a concentrate-to-forage ratio of 1:1. This consistent feeding regime stabilized rumen substrate supply, microbial community structure and ruminal pH, and minimized confounding effects derived from dietary variation on ruminal mycotoxin biotransformation. In the present study, the standardized feeding regimen maintained near-neutral ruminal pH, which favors β-reduction pathways over α-reduction. In addition, α-ZOL undergoes extensive hepatic first-pass clearance after absorption, resulting in negligible systemic accumulation. Meanwhile, β-ZOL persists longer in the rumen and circulation and thus becomes the major detectable metabolite in sheep. This pattern is consistent with findings in goats and cattle [21,28]. Gruber-Dorninger et al. [18] found that α-ZOL was the major metabolite in the rumen of lactating dairy cows, whereas both α-ZOL and β-ZOL were detected in feces. Kennedy et al. [29] reported that ZEN could be sequentially reduced to α-ZOL and β-ZOL in cattle, and α-ZOL was further metabolized into α-ZAL. This metabolic pathway was consistent with our results. This finding indicated that the ZEN reduction pathway was comparable between cattle and sheep, while metabolite profiles varied with species and test substrates.

ZEN exhibited the shortest elimination half-life, while β-ZOL and α-ZOL were eliminated at a relatively slower and comparable rate. Non-compartmental analysis (NCA) was adopted for rumen kinetic analysis in this study. The concentration data collected from 24 h to 48 h were selected as the terminal elimination phase for linear regression, with the coefficient of determination (R^2^) greater than 0.85. β-ZAL showed a bimodal concentration profile with two peaks appearing at 1 h and 24 h, so no valid terminal elimination phase could be defined and its elimination half-life could not be calculated. Similarly, Z14G exhibited low overall concentrations with drastic fluctuations in the rumen, and thus failed to obtain an effective fitting result. The elimination half-lives of β-ZOL and α-ZOL ranged from 10 h to 12 h, which were much longer than those of ZEN (7.44 h). This indicated that ZOL metabolites possessed higher stability in the rumen. Dänicke et al. [30] demonstrated in pigs that the elimination half-life of ZEN was shortened from 2.63 h to 1.1 h upon interruption of enterohepatic circulation, indicating that enterohepatic circulation can significantly prolong the gastrointestinal retention of this class of toxins. Catteuw et al. [15] also reported a secondary plasma concentration peak of ZEN glucuronide conjugates at 2 h after administration in piglet toxicokinetic studies, which is a bimodal feature typical of enterohepatic circulation. These findings suggest that ZOL metabolites may undergo sustained accumulation in the digestive tract, consistent with the markedly longer elimination half-lives of β-ZOL and α-ZOL compared with ZEN observed in the present study. Guo [25] further reported that the inhibitory effect of ZEN on rumen fermentation was more pronounced under high-roughage conditions, and that the degradation rate of ZEN increased with increasing dosage, which may also contribute to the differential elimination rates among metabolites.

Z14G exhibited a higher F_rum_ value than ZEN and β-ZOL. This finding indicated that although Z14G had the lowest absolute concentration and exposure in the rumen, it exhibited the highest efficiency of entering the systemic circulation after ruminal elimination. Gratz et al. [9] observed similar results in the Caco-2 cell model. Their data revealed that Z14G was barely absorbed by cells, with 91–93% recovered from the apical side of cells. However, its hydrolysate ZEN could be efficiently taken up and further metabolized. Lu et al. [12] calculated that the oral bioavailability of Z14G in rats was only 2.11%. Meanwhile, they pointed out that when the exposure levels of ZEN and its reductive metabolites were taken into account, the actual systemic exposure contributed by Z14G was far greater than that of the parent compound itself. Thus, despite low ruminal exposure, Z14G exhibited strong systemic conversion potential following hydrolysis and constituted a remarkable source of in vivo toxin exposure.

Feces and urine are the two primary routes for the elimination of drugs and their metabolites in animals. In this study, fecal and urine samples were continuously collected over 168 h to evaluate the excretion characteristics of Z14G and its metabolites. The apparent non-fecal recovery of Z14G in sheep reached 99.10%, and the detected free metabolites were primarily excreted via feces (accounting for 0.90% of the intake) and to a lesser extent via urine (0.04%). ZEN, α-ZOL, and β-ZOL were the predominant forms excreted in feces, whereas ZEN and β-ZOL were the major components detected in urine. Z14G itself was not detected in either feces or urine, confirming that Z14G undergoes rapid and complete hydrolysis in vivo.

This excretion pattern differs markedly from those reported in other animal species. Lu et al. [12] administered Z14G (10 mg·kg BW^−1^) via oral gavage to rats, and Z14G, ZEN, α-ZOL and β-ZOL were detectable in urine and feces starting at 6 h post-administration. Urine was identified as the primary excretion route for Z14G in rats, with peak concentrations of Z14G, ZEN, and α-ZOL reaching 2010–2170 ng·mL^−1^, 1030–1550 ng·mL^−1^, and 520–680 ng·mL^−1^, respectively. In feces, the concentrations of the three main metabolites also reached 330–770 ng·mL^−1^ before 48 h, all of which were significantly higher than those observed in sheep. Catteuw et al. [15,16] orally dosed piglets with Z14G at 0.331 mg·kg BW^−1^. No intact Z14G prototype was recovered in urine, and metabolites were predominantly excreted as glucuronide conjugates. The cumulative urinary recovery within 0–48 h post-administration reached 47%, indicating urine as the dominant elimination pathway. Although all above trials adopted Z14G as the test compound, feces served as the major excretion route in sheep, whereas urine prevailed in rats and pigs. Such discrepancy suggests fundamental interspecies differences in the gastrointestinal hydrolysis, absorption and excretion of Z14G.

Similar interspecies variations were also observed in studies with direct ZEN exposure. Dänicke et al. [30] intravenously injected ZEN into prepubertal gilts. Cumulative urinary excretion accounted for 70% of the administered dose within 72 h, while fecal excretion only accounted for 6%. Interruption of enterohepatic circulation reduced urinary and fecal excretion to 55% and 3%, respectively, and shortened the terminal elimination half-life of ZEN from 2.63 h to 1.10 h, indicating that enterohepatic circulation prolongs ZEN retention and modulates the distribution of excretion routes. Binder et al. [31] confirmed in pigs after oral ZEN administration that urinary recovery (26 ± 10%) was approximately twice that of fecal recovery (14 ± 3%) over 0–48 h. Dong et al. [21] detected ZEN, α-ZOL, and β-ZOL in both feces and urine of goats following intravenous ZEN injection, with β-ZOL as the predominant metabolite. Fecal metabolites existed mainly in free form, whereas urinary metabolites occurred almost entirely as glucuronide and sulfate conjugates. Qu et al. [22] administered ZEN orally to donkeys and detected ZEN, α-ZOL, and β-ZOL in both urine and feces, with β-ZOL excretion being higher than that of ZEN. Collectively, these findings indicate significant species differences in the excretion routes, metabolite profiles, and concentration levels of Z14G/ZEN among different animal species. Urine is the major elimination pathway in pigs and rats, while feces dominate metabolite excretion in ruminants such as sheep and goats.

Notably, the total metabolites recovered in feces and urine in this study accounted for only about 0.94% of the intake, which was substantially lower than values reported elsewhere (e.g., 47% urinary recovery in piglets after oral Z14G [15]). This discrepancy may partly reflect differences in distribution and excretion kinetics between oral and intravenous administration. It may also arise from the fact that the analytical method employed here did not capture all excretory forms. Multiple studies have confirmed that ZEN and its metabolites are predominantly eliminated as phase II conjugates, including glucuronides and sulfates. Dong et al. [21] verified that nearly all urinary metabolites existed in conjugated forms in goats, with free metabolite concentrations frequently falling below 10 ng·mL^−1^ and thus undetectable. Binder et al. [31] (oral ZEN in pigs) and Catteuw et al. [15,16] (oral Z14G in piglets) both identified ZEN-14-glucuronide, rather than free metabolites, as the primary urinary excretion marker. Sun et al. [13] further identified this conjugate as a highly abundant and continuously accumulating metabolite in rat plasma. In the present study, samples were not subjected to enzymatic hydrolysis, and the metabolites measured were exclusively in their free forms. Total excretion may therefore have been underestimated. Moreover, the 99.10% apparent non-fecal recovery was indirectly estimated from fecal recovery and may not fully account for microbial degradation, enterohepatic circulation, or other losses. This represents a limitation of this study.

Given the species-specific excretion patterns, the terminal tissue residue profiles of Z14G metabolites were further characterized in sheep.

At 168 h post-administration, ZEN was detected in all examined tissues, but the residue concentrations had decreased to low levels (1.38–3.61 ng·g^−1^). This indicates that ZEN formed from Z14G after conversion in sheep was widely distributed in tissues, but residue levels were significantly reduced after 168 h of elimination. Lu et al. [12] administered Z14G to rats and detected intact Z14G in gastric tissues. Its hydrolysate ZEN was widely distributed in various tissues, with the highest concentrations observed in the gastrointestinal tract. The study further identified widespread tissue occurrence of metabolites such as α-ZOL with potent estrogenic activity. Ruan [14] observed widespread distribution of ZEN and α-ZOL in the ovary and liver of rats following oral Z14G administration, and the ovarian and hepatic toxicity indicators in the Z14G group were significantly higher than those in the equimolar ZEN group. That study further demonstrated that Z14G could form self-assembled supramolecular gels in vivo, resulting in sustained release of ZEN and depletion of cytochrome P450, thereby eliciting stronger tissue toxicity than direct ZEN administration. In addition, Catteuw et al. [15,16] and Biehl et al. [17] provided evidence for enterohepatic circulation of ZEN, which can prolong its retention in the body and delay its excretion. These findings differ from the present study in that the parent compound Z14G was not detected in any sheep tissue. Nevertheless, the widespread distribution of ZEN across multiple tissues in sheep is consistent with the literature, indicating marked interspecies differences in the distribution and metabolic elimination of Z14G.

In the present study, the relatively higher residue concentrations of ZEN in the heart and lung may be attributed to the greater blood flow to these organs. Dong et al. [21] detected α-ZOL (5.2 ng·g^−1^) and β-ZOL (4.5 ng·g^−1^) in the liver of goats at 48 h after intravenous injection of ZEN, whereas ZEN itself was not detected. In the present study, ZEN was detected in the liver at 168 h (1.38 ng·g^−1^), but no ZOL metabolites were identified. This discrepancy may be related to differences in sampling time points (168 h vs. 48 h) and routes of administration (oral vs. intravenous). In addition, species-specific differences in the metabolic rate of ZEN and its reductive metabolites in the liver may also contribute to the observed disparity [20]. It is noteworthy that ZEN was detected in both the testis and epididymis, the male reproductive organs. Liu et al. [32] reported that ZEN could trigger cell apoptosis, suppress cell proliferation and upregulate the expression of pro-apoptotic genes via endoplasmic reticulum stress and oxidative stress pathways in Hu sheep Leydig cells. Ruan [14] demonstrated that sustained release of ZEN from Z14G enhances reproductive toxicity. Although the residue concentrations of ZEN in the testis and epididymis were low in this study, long-term low-dose exposure may still pose potential hazards to male reproductive function given the strong estrogenic activity of ZEN and its metabolites.

The detection of ZEN residues in edible tissues at 168 h post-dosing provides reference data for evaluating the potential food-safety implications of Z14G exposure. The residual concentrations ranged from 1.38 to 3.61 ng·g^−1^ across the examined tissues. To estimate potential consumer exposure, we constructed a conservative exposure scenario based on the ZEN concentration measured in sheep liver (1.38 ng·g^−1^), the 95th percentile sheep liver consumption of 43.5 g·day^−1^ [33], and an adult body weight of 70 kg [34]. Under this scenario, the estimated dietary exposure to ZEN from sheep liver consumption was 8.57 × 10^−4^ μg·kg^−1^ BW·day^−1^, representing approximately 0.34% of the EFSA tolerable daily intake (TDI) of 0.25 μg·kg^−1^ BW·day^−1^ [28]. The estimated exposure from sheep liver would remain well below the TDI, although this scenario represents a simplified single-matrix assessment and does not account for cumulative dietary exposure from other food sources or co-occurring ZEN metabolites.

The experimental dose of 2 mg·kg^−1^ BW was substantially higher than the potential exposure to Z14G from contaminated feed. To place the experimental dose in the context of potential dietary exposure, we used the maximum Z14G concentration of 15.31 μg·kg^−1^ reported in feed products [5], which was detected in premixed feed from pig and poultry samples, and assumed that the entire daily diet was uniformly contaminated at this concentration. Under this hypothetical scenario, a 36 kg sheep consuming 2.0 kg of feed daily would have an estimated exposure of approximately 0.85 μg·kg^−1^ BW·day^−1^, approximately 2350-fold lower than the experimental dose. Therefore, the tissue-residue concentrations observed here should not be directly extrapolated to tissue residues expected under typical dietary exposure conditions. Nevertheless, ZEN was detected in multiple edible tissues 168 h after Z14G administration at this high experimental dose. This finding highlights the need for further studies using realistic feed exposure levels and multiple withdrawal time points to characterize tissue residue depletion.

In addition, the exclusive use of male fattening sheep, the single-dose design, and the limited sample size (*n* = 5) mean that female-specific endpoints, chronic exposure scenarios, and inter-individual variability were not addressed. From a regulatory perspective, Commission Recommendation 2006/576/EC (as amended) [35] sets guidance values exclusively for parent ZEN, whereas no formal guidance is specified for its modified metabolites. In contrast, the recently adopted Commission Recommendation (EU) 2026/1801 [36], which will replace 2006/576/EC with effect from July 2027, puts forward a ZEN-equivalent strategy based on relative potency factors to incorporate modified ZEN forms into feed risk assessment. This illustrates that masked mycotoxins such as Z14G are attracting growing regulatory and scientific attention. Future investigations focusing on enzymatic hydrolysis for the quantification of total conjugated metabolites, chronic exposure regimens, female sheep models, and multi-time-point tissue sampling will extend toxicokinetic findings for risk assessment of masked mycotoxins in ruminants.

## 4. Conclusions

This study systematically elucidated the absorption, distribution, metabolism, excretion, and tissue residue characteristics of Z14G in sheep. Following oral administration, Z14G was rapidly hydrolyzed to ZEN in the rumen, which was subsequently reduced by rumen microorganisms to a variety of zearalenol (ZOL) and zearalanol (ZAL) metabolites. Among these, β-ZOL exhibited the highest cumulative exposure and the slowest elimination in the rumen. In serum, Z14G, ZEN, and β-ZOL all reached peak concentrations rapidly. Z14G exhibited the shortest elimination half-life, whereas β-ZOL showed the longest half-life and the highest AUC, making it the predominant form during the terminal elimination phase. The apparent volumes of distribution of Z14G, ZEN, and β-ZOL were all substantially greater than the total body water volume of sheep, suggesting extensive tissue distribution. Fecal excretion constituted the major elimination route, with minimal urinary excretion. At 168 h post-dosing, only ZEN residues were detected in various tissues. Relatively higher levels were found in the heart and lungs, and it was also present in the testes and epididymides. In summary, Z14G itself has an extremely short residence time in the body, and its toxicological risk is primarily attributable to its conversion to ZEN. Although β-ZOL possesses relatively low estrogenic activity, its prolonged exposure in vivo warrants further risk assessment. This study provides critical data to support the refinement of risk assessment for glycosidic masked mycotoxins in ruminants. It should be noted that these findings were obtained from male sheep receiving a single oral dose, and only free metabolites were quantified in the present work.

## 5. Materials and Methods

### 5.1. Chemicals and Reagents

The standards of 100 μg·mL^−1^ α-zearalenol (α-ZOL, CDAA-S-290069-JA, CAS: 36455-72-8), β-zearalenol (β-ZOL, CDAA-S-290070-JA, CAS: 71030-11-0), α-zearalanol (α-ZAL, CDFP-8517.18-10-AN, CAS: 26538-44-3, ChironAS, Trondheim, Norway), β-zearalanol (β-ZAL, CDAC-35407, CAS: 42422-68-4), zearalenone (ZEN, CDDS-A17947400AL, CAS: 17924-92-4), and zearalanone (ZAN, CDAA-S-290099-JA, CAS: 5975-78-0) were purchased from ANPEL Laboratory Technologies Inc. (Shanghai, China). The standards of zearalenone-14-glucoside (Z14G, 1ST157582, CAS: 105088-14-0) and zearalenone-14-sulfate (Z14S, 1ST157581M, CAS: 1439328-85-4) were purchased from Alta Scientific Co., Ltd. (Tianjin, China). Dimethyl sulfoxide (DMSO, D8371, CAS: 67-68-5) and physiological saline (IN9000, 7647-14-5) were purchased from Solarbio (Beijing, China). Chromatographic grade acetonitrile (ACN, 047138-M1, CAS: 75-05-8) and formic acid (28905, CAS: 64-18-6) were purchased from Fisher Scientific, Vienna, Austria. Other analytical grade reagents were purchased from Beijing Chemical Reagents Company (Beijing, China). Deionized water used in the experiments was purified by a Milli-Q system (Merck KGaA, Darmstadt, Germany; resistivity > 18.2 MΩ·cm). Nylon membrane filters (0.22 μm) were purchased from ANPEL Laboratory Technologies Inc. (Shanghai, China). Sample analysis was conducted at the Institute of Agricultural Quality Standards and Testing Technology, Chinese Academy of Agricultural Sciences. Details of the adsorbent materials was consistent with those reported by Suo et al. [19].

### 5.2. Instrumentation

A liquid chromatography-tandem quadrupole linear ion trap mass spectrometer (AB SCIEX Triple Quad 6500+) equipped with an electrospray ionization (ESI) source was used in this study, purchased from AB SCIEX (Framingham, MA, USA). Chromatographic separation was performed on a Waters BEH C18 column (100 mm × 3.0 mm, 1.7 μm) (Milford, MA, USA) at a column temperature of 30 °C, with an injection volume of 5 μL and a flow rate of 0.30 mL·min^−1^. The mobile phases consisted of phase A (0.1% formic acid in water) and phase B (0.1% formic acid in acetonitrile), using a gradient elution program. Mass spectrometric analysis was conducted in negative ion mode with a segmented detector. The detailed gradient program of the mobile phase and the complete mass spectrometric parameters are shown in Table 7 and Table 8, respectively.

### 5.3. Experimental Design

Five healthy male fattening sheep (crossbred Black-headed Suffolk × Mongolian sheep), aged 5 months, with similar body weights (36.06 ± 1.21 kg) were enrolled in the study. The experiment was conducted at the Hanshan White Cashmere Goat Breeding Farm in Chifeng, Inner Mongolia Autonomous Region, China. Animals were selected based on apparent health status, age, body weight and good general health status with no clinical abnormalities. The sample size of five animals represents the minimum number considered feasible for this large-animal toxicokinetic study, considering the extensive serial sampling of multiple biological matrices, terminal collection of multiple tissues, animal-welfare considerations, and the practical constraints associated with large-animal experiments.

All sheep were housed individually in metabolic cages. The adaptation and the formal experimental periods each lasted seven days. During the experiment, all sheep received standardized feeding twice daily (08:00 and 15:00) with free access to water. The basal diet was free from detectable Z14G and ZEN contamination and consisted of 0.4 kg corn, 0.6 kg commercial concentrate, and 1.0 kg forage (a mixture of corn straw and mung bean straw). Consistent feeding conditions were maintained to eliminate dietary confounding factors.

Animal welfare was monitored throughout the experimental period, with attention to general health status, feed intake, and abnormal behavior or clinical signs. To minimize animal discomfort and stress, experimental procedures were performed by experienced personnel, and unnecessary handling and repeated weighing were avoided. The predefined humane endpoints were a body-weight loss exceeding 15% or persistent refusal to eat. No unexpected adverse events or clinically apparent abnormalities were observed in any of the five sheep during the study.

This study was approved by the Laboratory Animal Welfare and Animal Experimental Ethical Committee of China Agricultural University (Approval No. AW31015202-1-4). No experimental protocol was preregistered.

The Z14G standard was dissolved in DMSO to prepare a 10 mg·mL^−1^ stock solution, which was diluted with physiological saline to a 2 mg·mL^−1^ working solution before use. Each sheep received a single oral dose of the working solution at 2 mg·kg^−1^ BW via a gavage tube. After administration, the gavage needle and syringe were rinsed with 50 mL of physiological saline, and the rinse solution was also administered to ensure accurate dosing.

Blank blood, rumen fluid, feces, and urine samples were collected before the start of the experiment (0 h). Blood samples were drawn from the jugular vein at 10, 30, 60 min, and 3, 6, 12, 18, 24, 30, 36, 48, 60, 72, 84, 96, 108, 120, 132, 144, and 168 h after Z14G oral administration. Serum was obtained by centrifugation at 1300× *g* for 15 min. Rumen fluid was collected at 1, 3, 6, 24, 48, 72, 96, 120, 144, and 168 h post-dosing. Feces and urine were collected every 6 h. Feces weight and urine volume were recorded at each collection. After 168 h post-dosing, the sheep were euthanized by intravenous overdose of pentobarbital sodium. Tissue samples were collected from the heart, liver, lung, kidney, spleen, testis, and epididymis; and bile samples were also collected. All samples were stored at −80 °C and subjected to LC-MS/MS analysis for ZEN and its seven metabolites (ZEN, ZAN, α-ZOL, β-ZOL, α-ZAL, β-ZAL, and Z14S).

### 5.4. Sample Extraction and Mycotoxin Quantitative Analysis

#### 5.4.1. Sample Extraction

For solid samples (feces and tissue samples): Weigh 5 g of sample into a 50 mL centrifuge tube, and add 20 mL of ACN and 0.2 mol·L^−1^ phosphate-buffered saline (PBS) mixture. The mixture was extracted on a reciprocating vertical shaker for 30 min and then centrifuged at 9400× *g* for 5 min.

For liquid samples (serum, urine, rumen fluid and bile): Transfer 1.0 mL of sample into a 10 mL centrifuge tube, add 4 mL of ACN and 0.2 mol·L^−1^ PBS mixture. The mixture was extracted on a reciprocating vertical shaker for 30 min and then centrifuged at 9400× *g* for 5 min.

#### 5.4.2. Purification

A 3 mL aliquot of the supernatant was transferred into a 10 mL centrifuge tube containing 50 mg of anhydrous sodium sulfate and 100 mg of the multi-adsorbent material. The mixture was vortexed for 30 s and then centrifuged at 9400× *g* for 5 min. The supernatant was evaporated to dryness under a stream of nitrogen at 40 °C. The residue was reconstituted in 0.5 mL of the initial mobile phase, vortexed for 30 s, and filtered through a 0.22 μm nylon membrane filter before being subjected to analysis.

### 5.5. Method Validation

The LC-Qtrap-MS/MS method was validated for linearity, accuracy, precision and matrix effects following Commission Implementing Regulation (EU) 2021/808 [37]. Performance for accuracy and precision was assessed at three spiking levels (1, 5, and 50 μg·kg^−1^ or μg·L^−1^) with six replicates per level. Full matrix-validation data covering 12 matrices are provided in Table S1.

### 5.6. Statistical Analysis

Concentration–time curves of Z14G and its metabolites in serum and rumen fluid, as well as excretion profiles in feces and urine, were plotted using GraphPad Prism 9.4.1 (GraphPad Software, San Diego, CA, USA). The excretion amounts in each collection interval were calculated and summarized. Serum toxicokinetic parameters of Z14G and rumen kinetic parameters were calculated using non-compartmental analysis (NCA) in WinNonlin 8.3.5 software (Certara, Inc., Princeton, NJ, USA). The terminal elimination phase was estimated by linear regression of the terminal points (at least three) on the log concentration–time curve. All curves were based on the mean values at each time point. All concentration and excretion data are presented as mean ± standard deviation (SD), with *n* = 5 sheep. For tissue residue data, values below the LOD were excluded from mean calculations. Mean ± SD values were calculated using samples with concentrations above the LOD. When only one sample had a concentration above the LOD, the individual value was reported without SD.

## Figures and Tables

**Table 1:** Linear equations, correlation coefficients (R^2^), LOD and LOQ of Z14G and its metabolites.

Mycotoxins	Linear Equations 0.5 μg·L^−1^~100 μg·L^−1^	Correlation Coefficient (R^2^)	LOD (μg·L^−1^)	LOQ (μg·L^−1^)
ZEN	Y = 101,614.12X + 12,030.28	0.9991	0.10	0.3
ZAN	Y = 79,374.83X + 17,547.62	0.9992	0.09	0.3
Z14G	Y = 39,377.09X + 17,906.47	0.9992	0.21	0.5
Z14S	Y = 33,699.06X – 21,653.73	0.9990	0.18	0.5
α-ZOL	Y = 43,453.72X − 2603.65	0.9991	0.16	0.5
β-ZOL	Y = 35,786.26X − 4711.38	0.9990	0.14	0.5
α-ZAL	Y = 56,655.65X − 2006.18	0.9990	0.15	0.5
β-ZAL	Y = 41,585.91X + 3533.22	0.9991	0.15	0.5

**Table 2:** Toxicokinetic characterization of β-ZOL, ZEN and Z14G in serum of sheep after a single oral administration of Z14G (*n* = 5).

PK Parameters	Unit	β-ZOL	ZEN	Z14G
Oral dose of Z14G	mg·kg^−1^·BW			2.00
T_max_	h	0.50 ± 0.00	0.50 ± 0.00	0.50 ± 0.00
C_max_	ng·mL^−1^	3.01 ± 0.59	20.25 ± 2.55	2.15 ± 0.78
T_1/2Elim_	h	2.79 ± 0.92	2.91 ± 1.49	0.94 ± 0.31
AUC_last_	ng·mL^−1^·h	6.41 ± 1.16	16.78 ± 1.08	3.10 ± 0.61
AUC_INF_	ng·mL^−1^·h	8.21 ± 2.16	20.77 ± 1.64	3.42 ± 1.45
AUC__%Extrap_	%	20.10 ± 9.54	14.04 ± 9.62	12.20 ± 8.68
MRT_INF_	h	3.83 ± 1.10	2.64 ± 1.18	2.68 ± 1.73
Cl__F_	L·kg^−1^·h^−1^	222.64 ± 24.68	96.58 ± 7.63	412.12 ± 113.12
Vz/F	L·kg^−1^	972.91 ± 124.35	397.00 ± 175.26	1037.13 ± 194.93

Serum samples were determined for Z14G, ZEN, ZAN, α-ZOL, β-ZOL, α-ZAL, β-ZAL, and Z14S. T_max_: time to maximum concentration, C_max_: maximum concentration, T_1/2Elim_: elimination half-life, AUC_last_: area under the curve from time zero to the last measurable time point, AUC_INF_: area under the curve from time zero to infinity (observed), AUC__%Extrap_: percentage of AUC extrapolated from the last time point to infinity, MRT_INF_: mean residence time extrapolated to infinity, Cl__F_: total body clearance, Vz/F: apparent volume of distribution.

**Table 3:** Local kinetic parameters of Z14G and its metabolites in sheep rumen fluid (*n* = 5).

Parameter	Unit	β-ZAL	β-ZOL	α-ZOL	ZEN	Z14G
T_max_	h	3.00 ± 0.00	1.00 ± 0.00	1.00 ± 0.00	1.00 ± 0.00	3.00 ± 0.00
C_max_	ng·mL^−1^	21.71 ± 5.55	154.75 ± 55.66	42.30 ± 27.66	83.56 ± 33.36	4.16 ± 0.69
AUC_(0–72h)_	ng·mL^−1^·h	437.24 ± 219.39	778.85 ± 254.68	161.54 ± 76.20	568.02 ± 257.79	38.81 ± 27.08
K_el_	1·h^−1^	-	0.06 ± 0.02	0.06 ± 0.03	0.10 ± 0.03	-
T_½_	h	-	11.90 ± 3.33	10.60 ± 3.16	7.44 ± 2.31	-
AUC_%Extrap_	%	-	4.12 ± 2.91	15.02 ± 7.86	9.08 ± 6.70	-
AUC_(0–∞)_	ng·mL^−1^·h	-	809.59 ± 253.53	243.23 ± 58.92	757.15 ± 239.88	-

T_max_ (time to peak concentration in rumen), C_max_ (peak concentration in rumen), and T_½_ (elimination half-life in rumen) were observed values derived from the rumen fluid concentration–time curves. AUC_(0–72h)_: rea under the curve from 0 h to the last detectable time point, calculated using the trapezoidal rule. K_el_: terminal elimination rate constant, calculated via linear regression of the terminal phase (24–48 h) of the logarithmic concentration–time curve of ruminal fluid. The software adopted a fitting criterion of R^2^ > 0.85. AUC_%Extrap_: percentage of the extrapolated portion relative to AUC_(0–∞)_. AUC_(0–∞)_: extrapolated area under the curve to infinity, corresponding to AUC_INF_ calculated by the software. For α-ZOL, this parameter was determined by extrapolating the measured AUC_(0–72h)_ based on the proportion of the terminal phase.“-”: relevant parameters were not determined.

**Table 4:** Total exposure burden and apparent absorption efficiency of metabolites in sheep rumen fluid (*n* = 5).

Metabolites	ΣA_rum_ (×10^6^ ng·h)	F_rum_ (%)
β-ZAL	2.40 ± 1.21	-
β-ZOL	4.45 ± 1.40	1.08 ± 0.71
α-ZOL	1.34 ± 0.32	-
ZEN	4.16 ± 1.42	3.50 ± 1.47
Z14G	0.21 ± 0.15	10.74 ± 5.68

ΣA_rum_: total rumen exposure load, calculated as ΣA_rum_ = AUC_(0–72 h)_ × V_rum_, V_rum_ = 5500 mL. F_rum_: apparent absorption efficiency, calculated as F_rum_ = (AUC_serum_/AUC_rum)_ × 100, using AUC_last_ to ensure comparability among groups. This index reflected the efficiency of each component transformed into systemic exposure after elimination from the rumen. It was assumed that the metabolites detected in serum are all derived from rumen elimination load, and the physical adsorption loss of chyme is ignored. “-”: relevant parameters were not determined.

**Table 5:** Contents of metabolites in feces and urine after single oral administration of Z14G (*n* = 5).

Parameters	Mass (mg)	Amount of Substance (μmol)
Ram body weight (kg)	36.06 ± 1.21	
Oral dose of Z14G (mg·kg^−1^·BW)	2.00	
Z14G intake	72.12 ± 2.41	150.09 ± 5.02
Fecal excretion of ZEN	0.29 ± 0.05	0.90 ± 0.16
Fecal excretion of β-ZOL	0.17 ± 0.05	0.53 ± 0.17
Fecal excretion of α-ZOL	0.15 ± 0.03	0.46 ± 0.11
Fecal excretion of α-ZAL	0.02 ± 0.00	0.06 ± 0.11
Fecal excretion of β-ZAL	0.03 ± 0.00	0.08 ± 0.01
Total Z14G excretion through feces (%)	0.90 ± 0.14	1.35 ± 0.21
Apparent non-fecal recovery (%)	99.10 ± 0.14	98.65 ± 0.21
Urinary excretion of ZEN	0.02 ± 0.01	0.06 ± 0.02
Urinary excretion of β-ZOL	0.01 ± 0.00	0.02 ± 0.01
Total Z14G excretion through urine (%)	0.04 ± 0.01	0.05 ± 0.02

Excretion amounts were calculated based on fecal sample weights, urine sample volumes, and the corresponding toxin concentrations measured in samples collected during the 0–12 h, 12–24 h, and 24–72 h intervals. Total Z14G excretion through feces (%) = (Total Z14G excretion via feces/Z14G intake) × 100. Total Z14G excretion via feces is the sum of ZEN, β-ZOL, α-ZOL, α-ZAL, and β-ZAL excreted in feces. Total Z14G excretion through urine (%) = (Total Z14G excretion via urine/Z14G intake) × 100. Total Z14G excretion via urine is the sum of ZEN and β-ZOL excreted in urine. Apparent non-fecal recovery (%) = ((Z14G intake − Total Z14G excretion via feces)/Z14G intake) × 100. This value is calculated solely from free metabolites recovered in feces.

**Table 6:** Toxin content in organs at slaughter (ng·g^−1^) (*n* = 5).

Organ	β-ZAL	β-ZOL	α-ZAL	α-ZOL	ZEN	Z14G	Z14S	ZAN
Testis	-	-	-	-	2.32 ± 2.63	-	-	-
Epididymis	-	-	-	-	3.05 ± 2.02	-	-	-
Heart	-	-	-	-	3.61 ± 2.64	-	-	-
Liver	-	-	-	-	1.38 ± 0.11	-	-	-
Lung	-	-	-	-	3.44 ± 2.86	-	-	-
Kidney	-	-	-	-	2.28 ± 2.01	-	-	-
Spleen	-	-	-	-	3.04 ± 2.97	-	-	-
Bile	-	-	-	-	1.65	-	-	-

Bile was collected as a biological fluid, and its concentration unit is ng·mL^−1^. “-” indicates concentrations below the LOD. Mean ± SD values were calculated using only positive animals. Values below the LOD were not included in the mean calculation. For bile, only one animal was positive. Therefore, the individual value (1.65) is reported without SD.

**Table 7:** Gradient conditions of mobile phase.

Time (min)	Flow (mL·min^−1^)	A: 0.1% Formic Acid Water (%)	B: 0.1% Formic Acid Acetonitrile (%)
0	0.3	90	10
1	0.3	90	10
2	0.3	75	25
3.0	0.3	70	30
3.1	0.3	10	90
5.0	0.3	10	90
5.1	0.3	90	10
8.0	0.3	90	10

**Table 8:** Mass spectrometry conditions for ZENMs.

Drug	Time (min)	Ion Par (*M*/*Z*)	Declustering Potential (V)	Collision Energy (eV)
ZEN	6.82	317.2 > 131.08 *; 317.2 > 175.1	100	35; 30
ZAN	4.45	319.0 > 205.1 *; 319.0 > 107.0	100	28; 34
Z14G	5.70	479.2 > 317 *; 479.2 > 175.1	120	10; 15
Z14S	6.11	397.1 > 317 *; 397.1 > 175.1	120	22; 15
β-ZOL	5.10	319 > 159.9 *; 319 > 174.2	120	32; 25
α-ZOL	5.44	319 > 159.9 *; 319 > 174.2	100	32; 25
β-ZAL	5.03	321 > 277.6 *; 321 > 161.2	120	10; 15
α-ZAL	5.36	321 > 277.6 *; 321 > 161.2	120	10; 15

* quantitative ion.

## Data Availability

The original contributions presented in this study are included in the article/Supplementary Materials. Further inquiries can be directed to the corresponding author.

## Supplementary Materials

The following supporting information can be downloaded at: https://www.mdpi.com/article/10.3390/toxins18090364/s1, Table S1: Matrix effect, intra-assay and inter-assay recovery and precision of ZEN-related mycotoxins across 12 sheep-derived matrices.
